# New York Letter

**Published:** 1895-07

**Authors:** 


### NEW YORK LETTER.

NEW YORK, June 27, 1895.

The medical world gives us little of interest during the summer months, but few medical societies holding their meetings between the last of May and the first of October. Much of the work ceases, and many are glad to get away after the winter grind. Teaching at the Post-Graduate School and the Polyclinic goes on, but is largely in the hands of the younger men, and a fresh advent of men, from the West and South mainly, come for instruction. Some, on their way to Europe, stop to look about, and decide to remain for awhile. They find plenty of opportunity for study at the clinics and the large hospitals and dispensaries. There is much for them to see and to do, and they are able, as a rule, to come into closer contact with instructors. Then, there is not the necessity of learning the language beforehand in order to get the full benefit of methods and instruction; whereas, unless a man is well grounded in French or German before starting abroad, at least six months there must be used in gaining but a scant acquaintance with a foreign tongue, though some few instructors there do make some use of English.

Following the changes that have been going on in the last six months in all the city departments, comes the separation of the Board of Charities and Correction into two new boards, and the new commissioners in charge of the hospitals belonging to the city already show a disposition to make some needed reforms. As is likely to follow at such times, some injustice may be done men who have done faithful work. The attending staff to the Harlem hospital has been retired, and it is rumored that the same thing will be done for the medical staffs of the City (formerly Charity) and Government hospitals. The appointing of new men will be divided between the College of Physicians and Surgeons, Bellevue, and University Medical College. If this is done, some recognition should be made of those men who have been faithful in the performance of their duties, by reappointing them. At the same time, it will not be an unmixed evil to get rid of a good deal of dead wood. It has been notorious that some men have clung to these positions, having the name of being members of the attending staff, but who have not been near the institutions for years, delegating other men to do their work for them.

The paper by Dr. White on "Result of Treatment of Hypertrophy of the Prostate" is on a subject that has been of considerable interest during the past year on account of the somewhat novel method recently recommended in some cases, and employed with success—that of double castration—and the distinct advance that has been made in the past year in successful removal of the gland itself. These two methods promise in the near future, to become rivals for surgical favor. Other methods proposed, as ligating the internal iliac arteries, will undoubtedly fall into oblivion. It is to be hoped that the near future will give some definite rule as to which may, in a given case, give the best prospect for a happy result. White presents, in his paper, the results collected from one hundred and eleven cases. The number of deaths following so comparatively simple an operation, twenty out of one hundred and eleven, or eighteen per cent., is startling, but of these twenty, White excludes twelve in estimating the mortality due to the operation *per se*, which leaves a mortality of seven per cent. This is certainly a higher percentage than we ought to expect in skillful hands; but he calls attention to the fact that in desperate cases in which death occurred, seventy-five per cent. showed shrinkage of the prostate before death.

He finds that 87½ per cent. of all the cases show rapid atrophy of the prostate following the operation. In 52 per cent. there was disappearance or great lessening of the cystitis present; in 66 per cent. there was a less disappearance of vesical dilatation; amelioration of most of the troublesome symptoms in 83 per cent; and that in 46 per cent. we may expect a return, to practically, a normal state. This certainly must be looked upon as an unmistakable showing; at the same time the personal bias, in favor of the operation, must be allowed for. We think the operation offers greater prospect of permanent return to nearly normal condition than any other form of treatment, when compared with the risks attendant upon other forms of treatment, as Prostatectomy. The idea that unilateras castration may prove sufficient is feasible in some cases; but at present, evidence on this point is contradictory. Two cases operated on in this manner resulted in marked improvement in the symptoms.

While Dr. White was reading his paper here, Dr. Eugene Fuller, of New York, read a paper entitled "Six Successive and Successful Cases of Prostatectomy" at the annual meeting of American Genitary-Urinary Association, held at Buffalo. In all six cases there was a complete recovery to normal condition in bladder symptoms, and complete return of an atonied bladder to normal.

The six operations were performed within the year. Dr. Fuller's method is to do a suprapubic cystotomy. The rectal bag is omitted, the patient lies flat, the Trendelenburg position is not used, the finger of the left hand seeks the internal meatus and the prostatic enlargement, and a pair of strong, long-handled scissors, with rough, serrated blades, are slipped along the finger and the mucous membrane of the floor of the bladder cut for an inch or more backward from the internal meatus, then with the doubled fist of one hand pushing up the perineum, the index finger of the other hand is inserted into the prostatic wound, and the enlargement is shelled out. Work is not stopped until all evidences of prostatic tissues have been removed. Then a perineal incision is made through the posterior urethra and into the prostatic wound, and a large perineal drainage tube inserted. The suprapubic wound is partially closed by deep catgut and superficial silkworm gut sutures, but the catgut suture is omitted in the middle of the wound and a silkworm gut suture taken through the entire thickness of the abdominal wall on either side, and through the bladder-wall, not including the mucous membrane. This is left loose, and a suprapubic drainage tube inserted, left in forty-eight hours or so, and on its removal the last suture is tightened.

The *Evening Post* of to-day, June 28th, contains the news of the resignation of Dr. Cyrus Edson as commissioner of the Board of Health. His resignaton having been asked for by Mayor Strong to hold in his possession, Dr. Edson goes further, and demands immediate acceptance of his resignation, to take effect July 1st. Dr. Edson has been a prominent figure in the Board of Health for a number of years, and has performed valuable and efficient work, and we have looked upon his retirement from his present position with regret.

